# Surface Electromyography-Based Recognition of Electronic Taste Sensations

**DOI:** 10.3390/bios14080396

**Published:** 2024-08-16

**Authors:** Asif Ullah, Fengqi Zhang, Zhendong Song, You Wang, Shuo Zhao, Waqar Riaz, Guang Li

**Affiliations:** 1 Institute of Intelligent Manufacturing, Shenzhen Polytechnic University, 4089 Shahe West Road, Shenzhen 518055, China; asifkh@szpu.edu.cn (A.U.); riazwaqar@szpu.edu.cn (W.R.); 2 State Key Laboratory of Industrial Control Technology, College of Control Science and Engineering, Zhejiang University, Hangzhou 310021, China; 12132035@zju.edu.cn (F.Z.);

**Keywords:** brain–computer interface (BCI), e-taste sensations and flavors, human–computer interaction (HCI), random forest classifier, surface electromyography(sEMG), taste recognition

## Abstract

Taste sensation recognition is a core for taste-related queries. Most prior research has been devoted to recognizing the basic taste sensations using the Brain–Computer Interface (BCI), which includes EEG, MEG, EMG, and fMRI. This research aims to recognize electronic taste (E-Taste) sensations based on surface electromyography (sEMG). Silver electrodes with platinum plating of the E-Taste device were placed on the tongue’s tip to stimulate various tastes and flavors. In contrast, the electrodes of the sEMG were placed on facial muscles to collect the data. The dataset was organized and preprocessed, and a random forest classifier was applied, giving a five-fold accuracy of 70.43%. The random forest classifier was used on each participant dataset individually and in groups, providing the highest accuracy of 84.79% for a single participant. Moreover, various feature combinations were extracted and acquired 72.56% accuracy after extracting eight features. For a future perspective, this research offers guidance for electronic taste recognition based on sEMG.

## 1. Introduction

Taste is one of the primary aspects of identifying food. People’s desire to consume different foods is primarily influenced by their sense of taste [1]. According to earlier studies, eating one’s favorite foods produces β-endorphins, a substance that aids in elevating mood [1]. According to the survey results, consumers primarily base their meal decisions on how the food tastes [2]. Although behavioral, social, and economic aspects influence dietary habits [3,4], customers claim that taste is the primary element influencing their diet decisions [2]. The basic definition of a food’s “taste” includes gustation, the oral sensation of composition, and the synthetic senses of taste [5,6,7]. Taste is essential to assess the excellence of food and mood activation [8]. Yet, different factors can cause taste disorders, such as anorexia, COVID-19 infection, cancer, and diabetes [9,10,11,12]. These disorders may be identified earlier by evaluating the patients’ taste perception. Hence, taste sensation is essential to assess disease diagnosis, digital taste, and various taste-related Brain–Computer Interfaces (BCIs) [13]

Recently, several researchers introduced a Brain–Computer Interface (BCI) based studies to investigate real taste sensations. This BCI includes magnetoencephalography (MEG) [14], electroencephalography (EEG) [14,15,16], electromyography (EMG) to examine how human facial muscles respond to tastes [17], and functional magnetic resonance imaging (fMRI) [18], which have been used to study how taste preferences affect our bodies physically. McCabe et al. utilized a Gaussian process classifier to recognize fMRI images of participants with dual vision and taste perception to determine taste aversion with over 80% accuracy [19]. However, they only discussed unpleasantness, did not identify basic taste stimuli, and used visual perception as an auxiliary. EEG has been helpful for identifying brain cognition based on various fundamental sensations [20] and odor sensation detection, similar to taste sensation recognition [21,22]. The effects of flavor stimulation from delights like alcohol and delicious pastries, among other things, could potentially be studied using EEG [23,24]. Park et al. collected EEG stimuli from subjects in pleasant (0.3 mol/L sucrose solution or milk chocolate solution), unpleasant (0.15 mol/L sodium chloride solution or mustard solution), and neutral (water) taste stimuli and used a support vector machine for pattern recognition to obtain an average accuracy above 70% [25]. Abidi et al. used kurtosis, skewness, energy, and wavelet entropy to categorize eight-channel EEG triggered by cube sugar or lemon juice with 98% accuracy [26]. EEG, however, has problems with data acquisition, redundant data, an excess of channels, and computing difficulties [15,27].

However, to solve these issues, a surface electromyography (sEMG)-based approach was introduced to analyze the relationship between taste and muscle activities [28]. The sEMG signals are the electrical impulses recorded across the skin surface during muscle contractions. Gustatory information from tasting food has an undesirable impact on the suprahyoid muscle activity patterns during pharyngeal ingestion. This method could evaluate taste responses in infants and individuals with disabilities [29]. Cosmetics, clinical diagnoses, sports mechanics, and artificial electromyography management are a few examples of sEMG applications [30]. This non-invasive sEMG technique has lately gained popularity for diagnosing muscle fatigue [31] and could identify salivary gland activation and facial activity in individuals [32,33,34]. sEMG records neuronal electrical impulses generated by a muscle’s sensory neurons and filaments. Prosthesis control, speech, and gesture recognition substantially use sEMG devices [35,36,37,38]. Various studies investigated sEMG features during eating or swallowing. For example, Sato et al. recorded and evaluated the sEMG of subjects consuming 18 standard food gels (six flavors and three textures combined) [39], whereas Miura et al. examined the influence of food carbonation and temperature on sEMG during swallowing [40]. Manda et al. recorded the EMG of the posterior tongue while chewing solid, semi-solid, and soft food and analyzed free and effortful mastication [41]. The results demonstrated that chewing cycles increased considerably during the free and laborious mastication of solid food. In contrast, there was no significant distinction between free and harsh semi-solid and soft food mastication. However, whether swallowing or chewing, it cannot be regarded as muscle activity under gustatory stimulation. Taste perception only occurs when the taste substance remains on the tongue or in the oral cavity.

Electronic taste has received much attention lately. Beancounter is an application that allocates jellybeans based on data sensitivity and memory transmission, while tastescreen users can taste food by licking the computer display [42]. Several researchers used practical magnetic resonance imaging to study higher cognitive operations to examine the link between taste novelty, temperament, and appetite control [43]. Additionally, a few projects in the field of medicine dealt with electronic stimulation of the human tongue [44,45,46]. Cruz et al. stimulated the tongue thermally and revealed that changing the temperature of the tongue’s regions can elicit tastes [47]. Lawless et al. presented a metallic taste based on electrical and chemical stimuli and discovered that a mild electrical impulse to the tongue produces a metallic-like taste [48]. Talavera et al. claimed that salty and sour tastes, among others, may be influenced by the surrounding temperature and chemical transcriptional pathways [49]. Further research stated that warming and cooling the tongue causes it to produce sweet and sour feelings [50].

In contrast, several other researchers developed a tongue-warming interface that delivers fatty and sweet tastes [51]. Some researchers have generated various basic tastes [52] and flavors [53] based on tongue stimulation. Until now, researchers have either focused on the classification of real taste sensations or the generation of basic tastes and various flavors based on the electrical and thermal stimulation of the tongue’s tip. To the best of our knowledge, no previous investigation has been conducted concerning the recognition of electronic taste (E-Taste).

**Figure 1:**
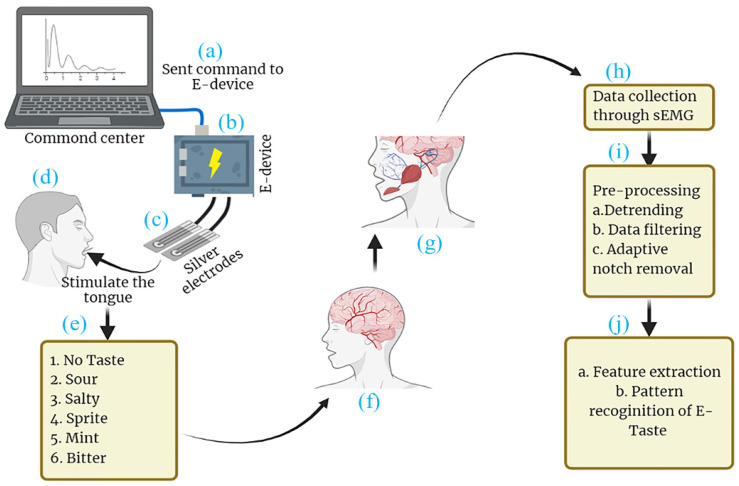
Schematic diagram of (a) command center, (b) E-Taste device, (c) tongue interface, (d) stimulating the tongue with E-Taste device, (e) generated tastes, (f) taste transmission to central nervous system (CNS), (g) CNS commanding muscles to activate, (h) data collected via sEMG, (i) the data being preprocessed, (j) features being extracted and E-Taste pattern being recognized.

This present study introduces a technique for the pattern recognition of E-Taste using surface electromyography (sEMG), as described in Figure 1. The primary objective is to record sEMG signals corresponding to six taste states: no taste, sour, sprite, bitter, mint, and salty. This research appeared to be the first attempt at conducting the pattern recognition of E-Taste. This study used a random forest classifier to fulfill the research goal, a well-known collective learning technique widely employed in pattern recognition applications. Before applying the classifier, the dataset was preprocessed, and various features were extracted [54,55,56].

The random forest classifier was trained and evaluated using a dataset comprising 13,440 samples and six labels representing the taste states. It is worth noting that the term “five-fold cross-validation accuracy” suggests that the dataset was divided into five subsets or folds, and the classifier was trained and tested five times, with each fold serving as the test set once. In contrast, the remaining folds were used as the training set. This study’s results indicated that the random forest classifier achieved an accuracy of 70.43% during the five-fold cross-validation process. This accuracy represents the classifier’s ability to correctly classify the taste states based on recorded sEMG signals. By achieving this accuracy level, this study accomplished its primary research objective of developing an E-Taste quality assessment approach based on sEMG signals.

Overall, this study introduces a novel technique for the pattern recognition of E-Taste using sEMG signals and demonstrates its feasibility by implementing a random forest classifier. The accuracy of the results suggests promising prospects for applying this approach in E-Taste quality assessment.

## 2. System Description

### 2.1. E-Taste Device

**Figure 2:**
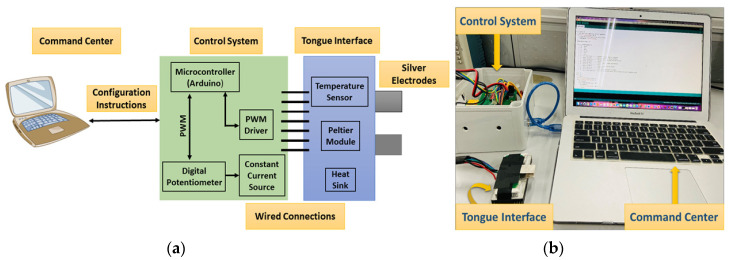
(**a**) Block diagram of E-Taste device and (**b**) implementation of E-Taste device [53].

The E-Taste device comprises the command center, control system, and tongue interface, as stated in Figure 2 [53]. An Arduino cable connects the command center to the control system. The command center instructs the control system to handle the output current and temperature. The control system comprises electrical and thermal sections.

The electrical stimulation comprises an Arduino, a digital potentiometer, and a constant current source (see Figure S1) to generate constant current pulses given to the silver electrodes with platinum plating (Ag/Pt). A digital potentiometer contains 256 taps with resistances of 10 kΩ, 50 kΩ, and 100 kΩ. The output current magnitude was regulated by manipulating the taps of the potentiometer through the Arduino connected to the command center (computer). The setting of the 256 taps was determined by the contact resistance linked to the constant current source. A comprehensive table has compiled all feasible tap configurations to achieve a particular current magnitude (refer to Table S1). Adjusting the tap values within the range of 55 to 215 results in a corresponding variation in the current magnitude, ranging from 40 µA to 180 µA, respectively, across the contact resistor linked to the constant current source. A constant current source is utilized to deliver a consistent flow of current, irrespective of the resistance of the tongue, which is known to vary among individuals. Throughout the experiment, a square-wave pulse characterized by a distinctive current spanning from 40 µA to 180 µA was delivered via Ag/Pt electrodes to the tip of the tongue. A square-wave pulse is preferred due to its ability to provide alternating (AC) and direct (DC) sensations. According to prior research, the frequency range for stimulating the tongue has been identified as 50–1000 Hz [50], and the average frequency for this study was 490 Hz.

The thermal section includes an Arduino and a DC motor driver (stated in Figure S2). The DC motor driver can drive the current path in two ways. A command center (computer) issues instructions to the Arduino microcontroller to operate the DC motor driver and heat the Peltier module placed on the surface of Ag/Pt electrodes up to 30 °C. Upon detecting a temperature of 30 °C, the microcontroller receives a command from a temperature sensor mounted to Ag/Pt electrodes. The microcontroller instructs the DC motor to modify the current path, thereby enabling the Peltier module to achieve a cooling effect of 20 °C with the assistance of a heatsink.

**Figure 3:**
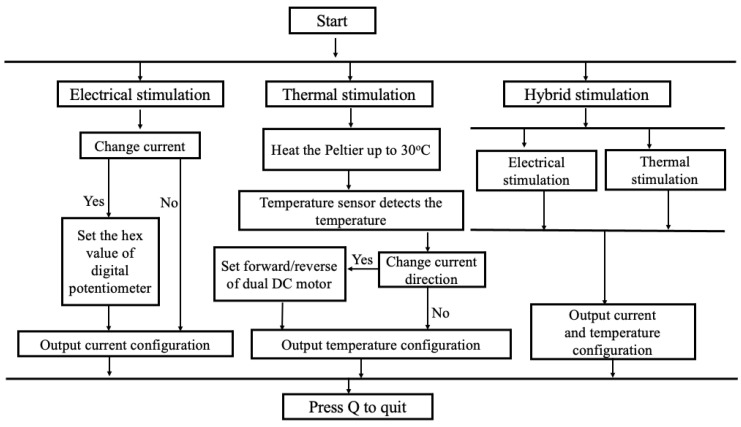
Flow diagram of E-Taste device.

The tongue interface is linked to the control system via connecting cables. The tongue interface comprises a Peltier module, a temperature sensor, two silver electrodes with platinum plating (5 cm × 1.5 cm × 0.02 cm), and a larger heatsink to sustain the cooling of the Peltier module (stated in Figure S3). Ag/Pt electrodes were used due to their excellent conductivity of electricity and temperature and non-causal metal sensitivity effect in humans. The flow diagram of the E-Taste is given in Figure 3.

### 2.2. Surface Electromyography

Surface electromyography is a technique to detect and analyze the electromyographic signals captured from the skin’s surface after muscle activations from undertaking some physical activity. The sEMG device employed in this study contains ten electrodes (six channels), among which there are two differential ones (channels 1 and 2), four single electrodes (channels 3, 4, 5, and 6), one reference (R), and one bias electrode (B). The sampling frequency of the sEMG is 1000 Hz. The electrodes are circular and have a 1.05 cm diameter with a sticky ring’s diameter of 1.5 cm.

## 3. Experimental Procedure

**Figure 4:**
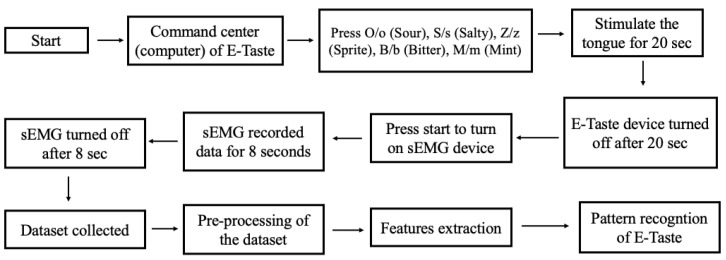
Flow chart of the pattern recognition of E-Taste.

Seventeen participants took part in the experiment. There were no taste disorders throughout the investigation, and all participants were healthy. Zhejiang University’s Ethical Board approved the human study for Animal and Human Safety. The flow diagram of the pattern recognition of E-Taste is given in Figure 4.

### 3.1. Validation of E-Taste Device

At first, the E-Taste device was tested on all the participants to generate various tastes and flavors. Three simulations were given to the tip of the tongue: electrical, thermal, and hybrid (electrical and thermal) stimulations [53]. The Ag/Pt electrodes were placed on the tip of the participant’s tongue. During the stimulation, various participants reported different tastes and flavors, among which 13 participants registered a sour sensation while giving the electrical impulse from 60 to 180 µA. By applying the current magnitude from 40 to 70 µA, 12 participants felt the salty sense, and 10 participants felt bitterness within 60 to 140 µA. During thermal stimulation, a few participants testified slight sweetness when the temperature was increased to heat the Ag/Pt electrodes. While decreasing the temperature below 25 °C, 13 participants sensed minty feelings. In total, 12 participants perceived the sprite taste during the hybrid stimulation. Some participants felt 3 to 5 taste sensations, and others felt only sour. Based on this observation, we chose 8 participants between 23 and 27 years old to collect the data through sEMG who sensed all the tastes mentioned in Table 1.

### 3.2. sEMG Acquisition System

Electrodes of the sEMG device are placed on the surface of the facial skin to capture signals (See Figure S4). The signal is processed by an embedded system comprising a low-pass filter circuit that has a cutoff frequency of 4000 Hz, an operational amplifier, and an analog-to-digital conversion module. Subsequently, the signal is transmitted to the host computer through a microprocessor and a Wi-Fi communication module. The signal’s sampling frequency is 1000 Hz. LABVIEW software, designed for human–computer interaction, was installed on the computer to record the signal.

### 3.3. Placement of Electrodes

**Figure 5:**
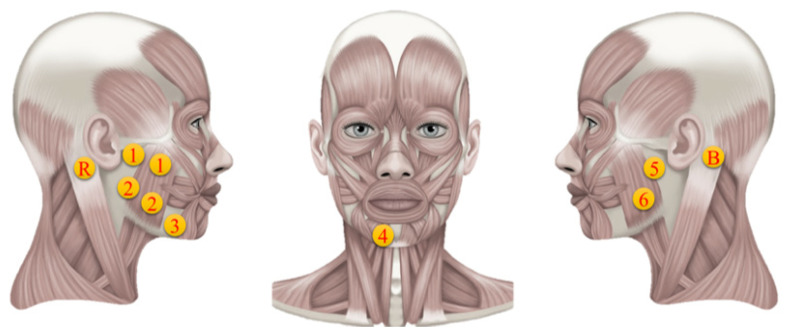
Electrode’s position on facial muscles [58].

The E-Taste device’s Ag/Pt electrodes were placed on the tongue’s tip. In contrast, the silver electrodes of the sEMG device were placed on facial muscles, which include the depressor angulus and depressor labium of the parotid glands [57], as indicated in Figure 5. The parotid gland is associated with salivation, while the muscles are involved in the emotional reaction to tasting stimulation. Previous research proved that taste stimuli could improve the depressor anguli oris and face muscles [17]. The behavior of the depressor anguli oris, which is controlled by the facial nerve, is further described by the depressor labium.

The acknowledged significance of these muscles in taste perception and facial muscle responses led to the placement of sEMG electrodes. Channels 1 and 2 were positioned on the right of the facial salivary area, while channels 5 and 6 were placed on the left side of the facial salivary region. Channel 3 was placed on depressor anguli oris, while channel 4 was positioned on depressor labium. The bias and reference electrodes were placed behind the left and right ear mastoid bones, respectively. A set of differential electrodes had an interlinked trail parallel to the direction of the fibers in the subsurface facial muscle. The two electrode sticky rings were spaced 1.5 cm apart in the middle. The reference electrode is conceivable for unipolar analysis, while the bias electrode reduces high-frequency deviation by reversing and enhancing the feedback signal. Table 2 provides the exact position of all the electrodes since the electrodes’ setup may affect the response, and the facial size of each participant is different [59].

### 3.4. Data Collection

The participant was prohibited from eating for two hours or consuming irritating foods 24 h before the experiment. There were 90 experiments, each including 4 sessions, and each session consisted of six trials (six taste sensations). In total, 360 sessions were carried out, including 13,440 samples (2520 samples per taste). The following steps were followed in the data collection process for a single session.

1. The E-Taste and sEMG devices were examined before the experiment began.
2. sEMG electrodes were placed on the participant’s face, as shown in Figure 5, while he was instructed to sit back and relax.
3. The participant was instructed to put the tongue’s tip between the Ag/Pt electrodes of the E-Taste device.
4. Various taste sensations were generated on the tongue’s tip, as mentioned in Table 1. The time duration of the tongue’s stimulation was 20 s.
5. sEMG signal was captured from the facial muscles for 8 s after generating the taste on the tongue.
6. The participant was told to wash their mouth or drink water and take a one-minute break during each taste sensation.
7. After finishing all six taste sensations, the participant was instructed to take a five-minute break.
8. After the five-minute pause, another session was started.
9. All the above steps were repeated for each participant.

## 4. Preprocessing and Feature Extraction

The duration of sEMG data collection was 8 s per session per taste, and the stimulation duration of the tongue was 20 s. Sliding windows cut the sEMG data into 1 s sample lengths to enhance the model.

### 4.1. Data Filtration

Generally, sEMG signals are susceptible to the following interferences: First, the Ag-AgCl wet electrode, which is commonly utilized, exhibits a high signal-to-noise ratio. Nevertheless, it exerts a distinct impact on the signal, such as the low-pass effect [60]. The second aspect pertains to mobility artifacts, typically associated with the surface electromyography (sEMG) response resulting from the electrode–skin movement, commonly observed within the frequency range of 1–10 Hz [61]. The phenomenon may also pertain to the interference resulting from the potential difference between the skin and the electrode. The application of conductive gel between the electrode and the skin of the wet electrode, along with the pre-experiment cleansing of the face, can reduce impedance. This impedance reduction can minimize such an interference’s influence [62]. Thirdly, “electromagnetic interference” pertains to the impact of electromagnetic signals emanating from the ambient surroundings on surface electromyography (sEMG) obtained from the human body or through acquisition equipment. Implementing common-mode suppression can significantly reduce this interference [63]. The most common type of electromagnetic interference is power frequency interference. The interference is due to the 50 Hz alternating current that powers electronic devices in the surrounding environment. The most common type of power frequency interference manifests as signals that operate at 50 Hz and their harmonics. Lastly, sEMG signals are affected by internal noise, which can lead to alterations in skin and muscular properties [64].

These interferences caused by the movement of the articulator to the sEMG can be seen through the measurement of the experimental signal, primarily in the baseline drift and the evident change in the signal amplitude range. The movement of the articulator caused this interference. It is difficult to effectively isolate the numerous interference signals, the low-pass effect, and the interaction of electrodes; however, it is possible to keep it stable by controlling the experimental process, such as electrode position and inter-electrode distance (IED), etc. It is carried out to make it as consistent as possible so that it does not affect the pattern recognition process that comes after it. The influence of skin and muscular characteristics on the signal cannot be eliminated nor addressed; however, it can be mitigated by shortening the duration of the experiment and ensuring that the participant’s physical and mental states remain stable throughout the investigation. In short, the interference has three manifestations in the signal: baseline drift (caused by inherent noise of the electrode, motion artifact, or articulator movement, etc.), power frequency interference, and abnormal amplitude range (caused by excessive power frequency interference or articulator movement, etc.). During the preprocessing, the baseline drift is removed by eliminating the trend item combined with high-pass filtering, the adaptive power frequency notch removes the power frequency interference, and the samples with abnormal signal amplitude are removed by abnormal sample removal. The following steps were taken to address the problems mentioned earlier.

**Figure 6:**
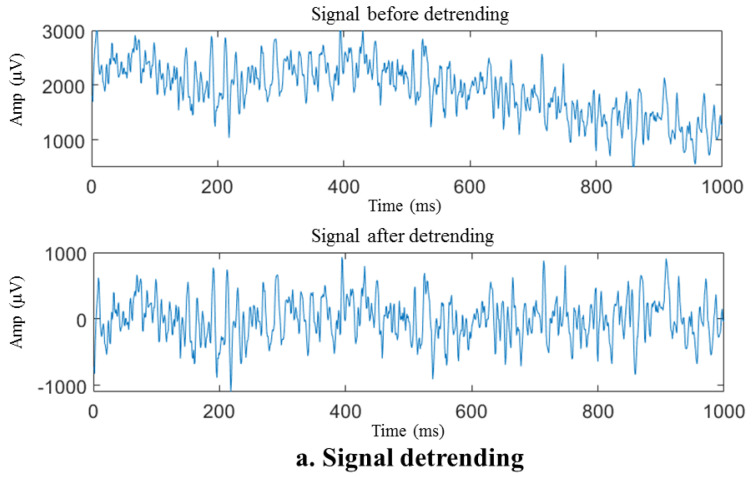

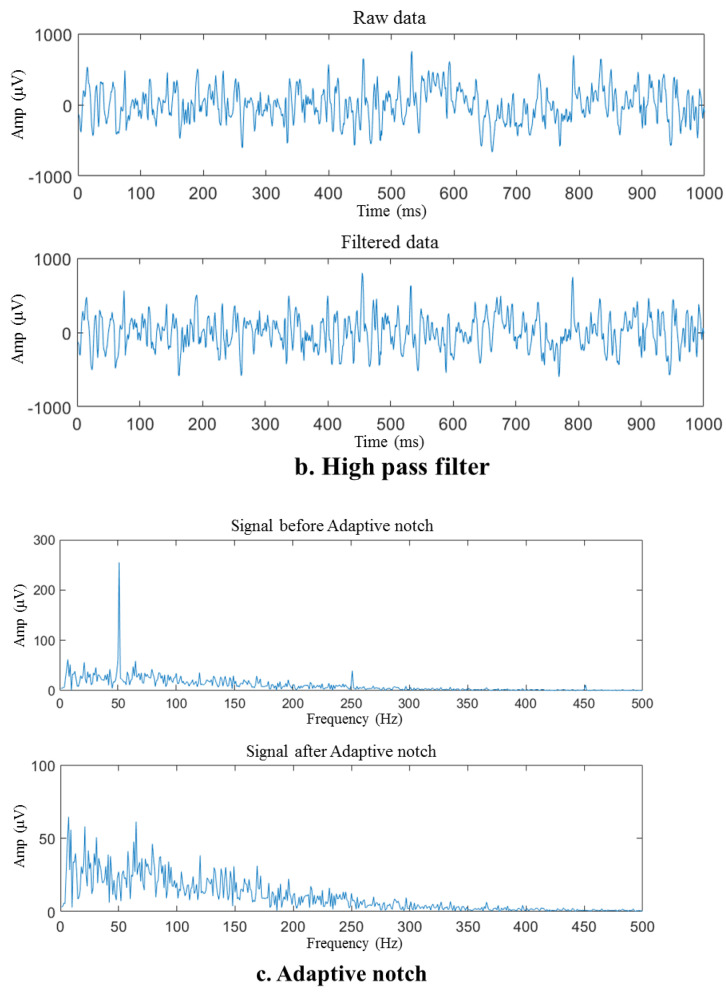
Preprocessing of the sEMG sample signal. (**a**) Signal before and after detrending, (**b**) raw and filtered data, (**c**) signal before and after adaptive notch.

Baseline drift removal, also known as baseline correction, is a two-step procedure. Firstly, observing the signal characteristics reveals that the baseline of the sEMG sample waveform with a duration of 1 s can be roughly defined by a polynomial curve with a degree no greater than four. To mitigate the baseline drift, the sEMG sample waveform item undergoes removal of a fourth-degree polynomial trend, as depicted in Figure 6. Subsequently, the signal undergoes processing via a fourth-order Butterworth filter, employing a cutoff frequency of 10 Hz because the motion artifacts produced by baseline drift are mainly concentrated within the 1–10 Hz range. Butterworth filters are extensively used in signal processing due to their maximal passband flatness [65].

The removal of the polynomial trend components, as illustrated in Figure 6a, results in a significant reduction in the DC component and baseline drift of the signal, enabling the fluctuation range of the signal to be restored to a reasonable range (within about 1000 μV for this acquisition system). The energy in the low-frequency near-DC portion depicted in Figure 6b that reaches the millivolt (mV) level is removed. However, seeing the difference in waveform before and after the high-pass filter is complex. Despite detrending the signal, some spikes in the spectrum still cannot be ruled out due to movement artifacts. After applying a high-pass filter, the spikes mentioned above were eliminated, thereby signifying the efficacy of the high-pass filter.

After detrending and high-pass filtering, the sEMG waveform sample underwent processing via an adaptive notch filter to remove the 50 Hz fluctuation and its harmonics that arise from power frequency interference, as illustrated in Figure 6c. The self-adaptive notch filter is designed to modify the notch intensity of a frequency point by utilizing the average signal amplitude of the current frequency spectrum at the power frequency or its frequency multiplier and the frequency spectrum amplitude at the adjacent interval. The signal spectrum after baseline correction includes unusual peaks that are concentrated at frequencies such as 50 Hz and 250 Hz, indicating power frequency interference. The notch filter effectively removed these spikes, revealing that the adaptive notch filter can minimize the influence of filtered-out power frequency interference.

As previously stated, after removing baseline drift and power frequency interference, signals with abnormal amplitudes must also be removed. We set criteria to eliminate abnormal signals. That is, each channel of each sEMG sample signal is considered a standard signal if the maximum value of the absolute value of the signal does not surpass 2000 μV; that is, the fluctuation range of the sample signal does not exceed −2000 μV to 2000 μV (an example of a standard signal is stated in Figure S5). The signals on the right side of Figure S5 range between −2000 μV and 2000 μV, while the signals beyond this limit were discarded.

### 4.2. Features Extraction

Twenty-one features were extracted from the sEMG data, among which sixteen were frequency-domain-based while five were time-domain-based features. In total, 126 features were retrieved because there were six channels and 21 different features. Table 3 provides a list of 21 dimension features for each channel.

**Figure 7:**
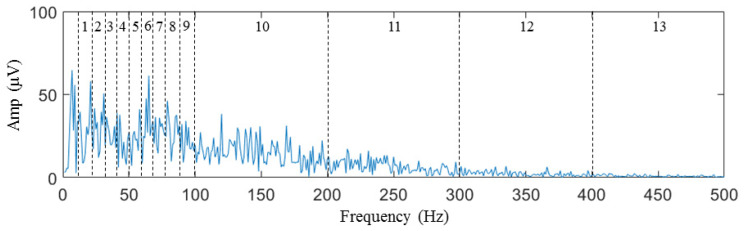
Interval division of features from 1st to 13th.

During the sEMG data collection after generating the taste on the tongue, the characteristic variations in the signal in the time domain are not readily apparent. However, the information in the frequency domain is of greater significance. The most specific information about a signal in the frequency domain is the spectrum. The average interval amplitude of the spectrum is used as the feature in the spectrum histogram concept to investigate the energy distribution of the signal in the frequency domain. The spectrum is divided into 13 frequency intervals for every channel, as shown in Figure 7. The average intensity of the spectrum in each frequency interval is determined as a feature that can be used to classify the spectrum. Since the signal went through initial high-pass filtration utilizing a 10 Hz cutoff frequency, the frequency spectrum segment below 10 Hz did not contain any resourceful information and was therefore excluded from further analysis. The concentration of energy in the sEMG sample spectrum was primarily observed below 100 Hz, which can be attributed to the low-pass filter introduced by the circular electrode [66].

Consequently, the size of the interval was established as 10 Hz, ranging from 10 to 100 Hz, resulting in a total of nine frequency intervals. In contrast, the interval size was set 100 Hz from 100 to 500 Hz, resulting in four frequency intervals. For each frequency interval, we calculate the average amplitude of the sample signal spectrum as a feature according to Equations (1) and (2).

Additionally, three frequency domains, including frequency centroid (FC), root mean square frequency (RMSF), and root variance frequency (RVF), and five time-domain features, including kurtosis (Ku), mean absolute value (MAV), root mean square (RMS), skewness (Kw), and zero-crossing rate (ZCR), were taken into account. In total, 21 different feature types were determined using the equations from 3 to 10 [28].

(1) $$F\left[n\right]=\frac{1}{10}\sum_{i=n\times 10}^{\left(n+1\right)\times 10-1}f\left(i\right),when\;1\leq n\leq 9$$

(2) $$F[n]=\frac{1}{100}\sum_{i=(n-9)\times 100}^{\left(n-8\right)\times 100-1}f\left(i\right),when\;10\leq n\leq 13$$

(3) $$FC:F[14]=\frac{\sum_{i=1}^{L/2}f(i)\times (i\times f_{s}/L)}{\sum_{i=1}^{L/2}f(i)}$$

(4) $$RMSF:F[15]=\sqrt{\frac{\sum_{i=1}^{L/2}f(i)\times {\left(i\times f_{s}/L\right)}^{2}}{\sum_{i=1}^{L/2}f(i)}}$$

(5) $$RVF:F[16]=\sqrt{\frac{\sum_{i=1}^{L/2}f(i)\times {\left(i\times \frac{f_{s}}{L}-F[14]\right)}^{2}}{\sum_{i=1}^{L/2}f(i)}}$$

(6) $$RMS:F[17]=\sqrt{\frac{\sum_{i=1}^{L}{x_{i}}^{2}}{L}}$$

(7) $$ZCR:F[18]=\frac{1}{10}\sum_{i=1}^{L-1}1,when\;x_{i}x_{i+1}<0$$

(8) $$MAV:F[19]=\frac{\sum_{i=1}^{L}\left|x_{i}\right|}{L}$$

(9) $$Ku:F[20]=\frac{1}{L}\sum_{i=1}^{L}{\left(\frac{x_{i}-µ}{\sigma }\right)}^{4}$$

(10) $$Sk:F[21]=\frac{1}{L}\sum_{i=1}^{L}{\left(\frac{x_{i}-µ}{\sigma }\right)}^{3}$$

F[n] denotes the nth feature. The frequency domain’s ith point’s spectrum amplitude is represented by the notation f(i). The length of the sample is indicated by L, which is 1 K milliseconds. The sampling frequency fs is 1 K Hz. xi is the ith data point’s value in the time domain. The mean value is represented by µ while σ is the standard deviation of the data.

## 5. Classification

Random forest is a classifier that uses many decision trees on different subsets of the input dataset and averages the results to increase the dataset’s predicted accuracy. At first, a random forest algorithm was applied to one participant’s dataset to demonstrate the identification problem’s viability. The features were chosen based on the performance of the random forest algorithm on datasets with various features’ significance. A total of 255 datasets containing every possible combination of eight participants were constructed to examine how participant diversity affected model performance. A random forest was used to compare the accuracy of models trained on various participant counts.

### 5.1. Results and Discussion

#### 5.1.1. Feature Selection

Features were selected and extracted based on the prior studies and the feature’s significance. Shi et al. used sEMG to collect the data from lower limb motion and extract MAV, RMS, and WTC features to identify the lower limb motion [67]. Wang et al. used sEMG to analyze the relationship between real taste (sugar, vinegar, coffee, etc.) and facial muscle response and extracted various time and frequency domain features [28]. Zecca et al. extracted mean absolute value (MAV), mean absolute value slope (MAVS), zero crossings (ZCs), Slope Sign Changes (SSCs), and Waveform Length (WL) to describe the control of a multifunction prosthesis [68]. They showed that the myoelectric signal exhibits a deterministic structure during the initial phase of a muscle contraction. Other researchers proposed a review about using sEMG to assess muscle fatigue by extracting various time and frequency domain features, including RMS and MAV [69].

Earlier studies mentioned that each feature provides specific information regarding the signal. For example, spectrum average amplitude gives information related to the signal amplitude at each frequency, the RMSF (root mean square frequency) of a spectrum is a single number that represents the overall level of energy across a frequency range, MAV defines the average of the summation of the absolute value of a signal, and a ZC counts the times that the signal changes’ sign, etc. Based on these facts, the features were selected to analyze the signal better.

**Figure 8:**
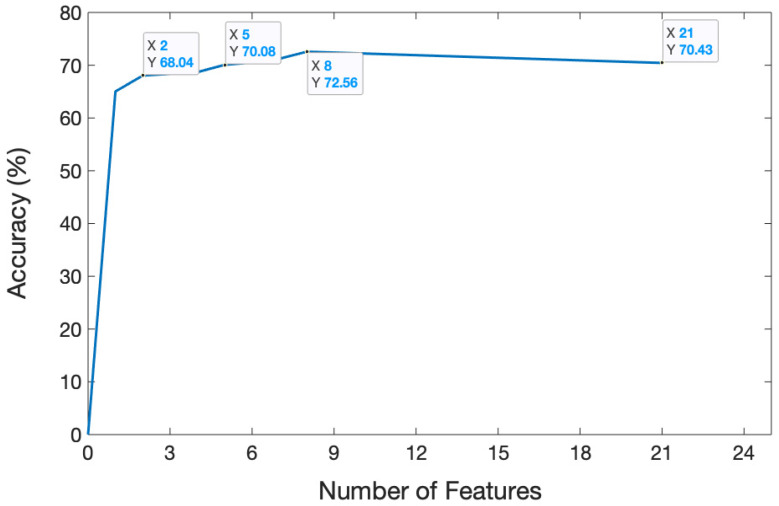
Relation between the number of features and accuracy. The graph illustrates how accuracy changed over time as the number of features increased.

**Figure 9:**
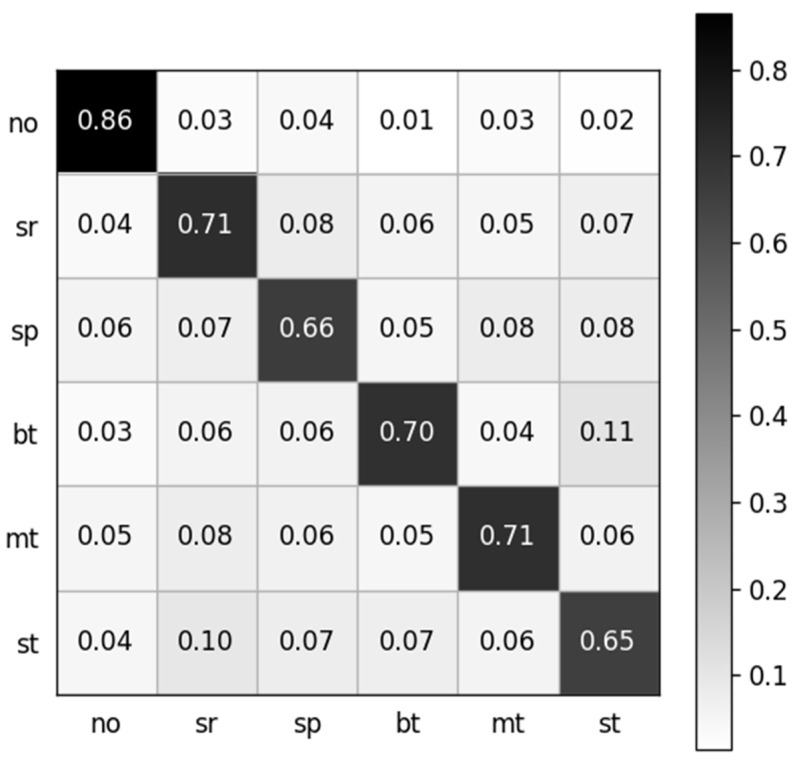
Normalized confusion matrix. The labels are denoted as follows: no: no taste, sr: sour, sp: sprite, bt: bitter, mt: mint, st: salty.

The dataset containing 13,440 samples was utilized to analyze the sEMG-based recognition of electronic taste sensation. Twenty-one features were extracted from the sEMG dataset using a random forest classifier. To analyze the data better, we extracted one feature, which gave a five-fold accuracy of 68.69%. After extracting two features, the accuracy decreased to 68.04%. The accuracy reached 70.08% after extracting five features. The curve hit its turning point at the eighth feature, and the model accuracy climbed to 72.56%. However, after extracting 21 features, the model gave a five-fold accuracy of 70.43%. The features were selected in random order. The relationship between the number of features and accuracy is shown in Figure 8, while the confusion matrix after extracting 21 features is depicted in Figure 9.

#### 5.1.2. Participant’s Grouping

In the next step, we achieved the results by grouping the participants. At first, the random forest was applied to a single participant dataset after extracting 21 features, and the accuracy was 84.79%. After using the random forest classifier on two participants’ datasets, the accuracy decreased to 82.57%. We added one participant’s dataset in each step to observe whether the accuracy increased or decreased. With the increasing number of participants, the accuracy became lower. Taste varies among individuals, and the decreasing accuracy could be the response of different participants’ facial muscles to various electronic stimulations of the taste sensation. The relation between participants’ grouping, multiple datasets, and accuracy is shown in Table 4. The results specified that the approach was suitable for each participant combination. A random forest model applied on a dataset comprising 13,440 samples from eight participants gave an accuracy of 70.43% for 21 features.

#### 5.1.3. Comparison of E-Taste and Real Taste Sensation

To validate the E-Taste recognition, we collected the dataset of both actual taste materials and electronic taste from three subjects for four taste states (no taste, sour, bitter, and salty). The dataset included 3920 samples for both actual and E-Taste sensations. All the steps mentioned above during the data collection of both E-Taste and actual taste stimulation were followed except the fourth step (iv). The tongue’s tip was stimulated with various current stimuli for E-Taste sensations to generate the taste. In contrast, sour (refined vinegar), bitter (instant coffee powder), and salty (refined salt) tastes were placed on the tongue one by one through a circle-shaped spoon with a 1.1 cm radius and capacity of 2 mL during the data collection of actual taste sensations. After placing the flavors on the tongue, the participant was asked to close their eyes and mouth, relax their mind, and not chew. The sEMG data collection duration was 8 s.

**Figure 10:**
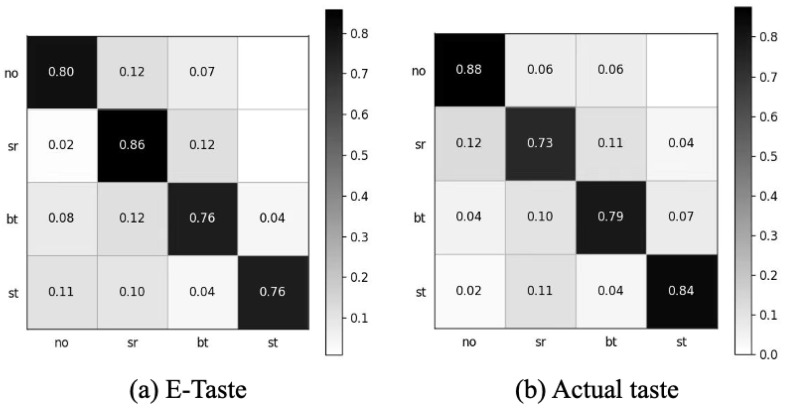
Confusion matrix of (**a**) E-Taste states and (**b**) actual taste states.

After obtaining the data, we employed the preprocessing (signal detrending, high-pass filter, adaptive notch removal, and signal removal with abnormal amplitudes) mentioned in this manuscript (Figure S6). We extracted the same 21 features for both E-Taste and actual taste sensations. The E-Taste stimulation gave a five-fold accuracy of 79.46%, and the actual taste gave a five-fold accuracy of 80.8%, which proves the validation of E-Taste recognition based on sEMG. Figure 10 depicts the confusion matrix for E-Taste and actual taste stimulation.

### 5.2. Limitations and Directions for Further Research

The investigation regarding the pattern recognition of E-Taste reveals a few shortcomings. Firstly, the E-Taste device is not user-friendly and is not suitable for regular use. Few participants hesitated to put the electrodes of the E-device on the tongue as they were anxious about burning their tongue or hygiene. Therefore, improving the technology would benefit from working with experts from the medical field. Secondly, various combinations of sEMG channels should be examined. We should analyze the performance of all six channels separately and in combinations and identify the combinations of the features. Thirdly, we need to explore further the combination of the features that best suit this study. Future studies should observe every possible combination of 21 features. Fourth, identifying the specific features that work best for the exact participants needs to be recognized. Fifth, a pre-validation of the pattern recognition of E-Taste by comparing it with the actual taste sensations has been realized. However, further investigation is needed to compare actual and E-Taste pattern recognition, including the five basic tastes. Sixth, the random forest classifier is a traditional method; applying several other machine learning methods, such as deep learning, could improve the accuracy. Finally, there were insufficient participants and an insufficient dataset; the model’s performance could be best defined with more participants and a larger dataset.

## 6. Conclusions

This study provided the viability of electronic taste recognition based on surface electromyography. An experimental approach was established to record the sEMG signals in response to several taste and flavor stimuli. The data were then compiled and analyzed using a random forest classifier. Twenty-one features were extracted one by one to observe the model’s performance. The next step was to examine the features grouping. At first, we extracted one feature and combined one more feature in each step. The accuracy for the two feature group was 68.04%, while eight features gave 72.56% accuracy. After extracting 21 different types of features, the model demonstrated the viability of this identification task with a five-fold cross-validation accuracy of 70.43%.

Additionally, we evaluated the model’s performance on various participants’ datasets, and this method was sustainable for each participant separately and in groups. However, the accuracy decreased with the increase in participants, which could be due to the response of the participant’s facial muscles to different taste stimuli. Several other reasons may decrease the accuracy, including electrode positions on facial muscles, residual noise, and artifacts.

Furthermore, a comparison of E-Taste with actual taste sensations was carried out to prove this study. This study’s primary objective was to suggest a unique technique for recognizing electronic taste sensations, providing a reliable method for training, analyzing, and classifying E-Taste using sEMG.

## Figures and Tables

**Table 1:** The range of electronic taste stimulation and the number of participants who identified various tastes and flavors.

Tastes and Flavors	Stimulation’s Range	Number of Participants Recognizing Taste Sensation
No Taste	Nil	
Sour	60–180 µA	13/17
Salty	40–70 µA	12/17
Sprite	60–180 µA and 30–20 °C	12/17
Bitter	60–140 µA	10/17
Mint	Below 25 °C	13/17

**Table 2:** Types of electrodes and their detailed positions on facial muscles.

Electrodes	Electrode Type	Muscle	Detailed Placement
1	Differential	Masseter	Below the right ear
2	Differential	Masseter	In line and beneath channel 1
3	Single	Depressor anguli oris	Mouth’s lower right corner
4	Single	Depressor labium	Under the lower lip
5	Single	Masseter	Below the left ear
6	Single	Masseter	In line and beneath channel 5
B	Bias	Mastoid bone	Behind left ear
R	Reference	Mastoid bone	Behind right ear

**Table 3:** Feature’s name, domain, and dimension.

Name	Feature’s Domain	Dimension
Spectrum average amplitude	Frequency	13
RVF	Frequency	1
FC	Frequency	1
RMSF	Frequency	1
MAV	Time	1
Sk	Time	1
RMS	Time	1
Ku	Time	1
ZCR	Time	1

**Table 4:** Relationship between subject’s dataset grouping and accuracy. The numbers from 1 to 8 show a group of participants, e.g., 5 means dataset containing 5 participants’ dataset.

Number of Participants	Dataset	Accuracy (%)
1	1680	84.79
2	3360	82.75
3	5040	80.61
4	6720	79.52
5	8400	75.36
6	10,080	72.82
7	11,760	71.49
8	13,440	70.43

## Data Availability

The dataset is available upon reasonable request.

## Supplementary Materials

The following supporting information can be downloaded at https://www.mdpi.com/article/10.3390/bios14080396/s1.
