# Supplementary material for: Surface Electromyography-Based Recognition of Electronic Taste Sensations
Source: Biosensors (Basel). 2024 Aug 16;14(8):396. doi: 10.3390/bios14080396 (PMC11352680; doi:10.3390/bios14080396)
Supplement: Supplementary file 1 [file biosensors-14-00396-s001.zip › biosensors-3110883-supplementary.pdf]

<u>Supporting Information</u>

Surface Electromyography-Based Recognition of Electronic Taste Sensations

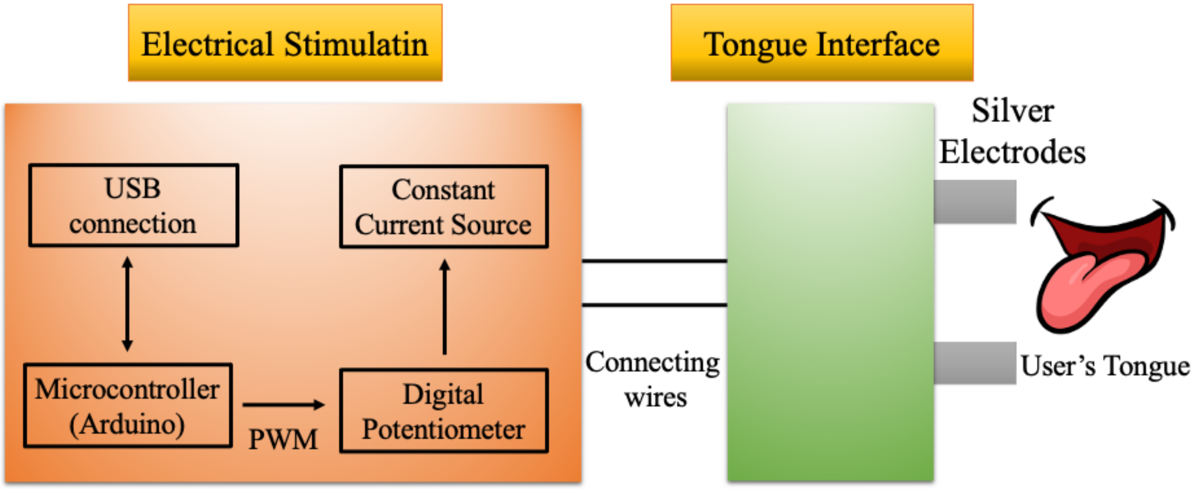

Figure S1. Electrical stimulation includes a digital potentiometer and a current source to provide a constant current to the tongue interface.

Table S1. Digital Pot values and corresponding output voltage and current values

POT values (i)	Voltage (V)	Current μA
10	0.28	14
20	0.43	21.5
40	0.74	37
60	1.02	51
80	1.28	64
100	1.54	77
120	1.82	91
140	2.12	106
160	2.42	121
180	2.8	140
200	3.24	162
220	3.78	189

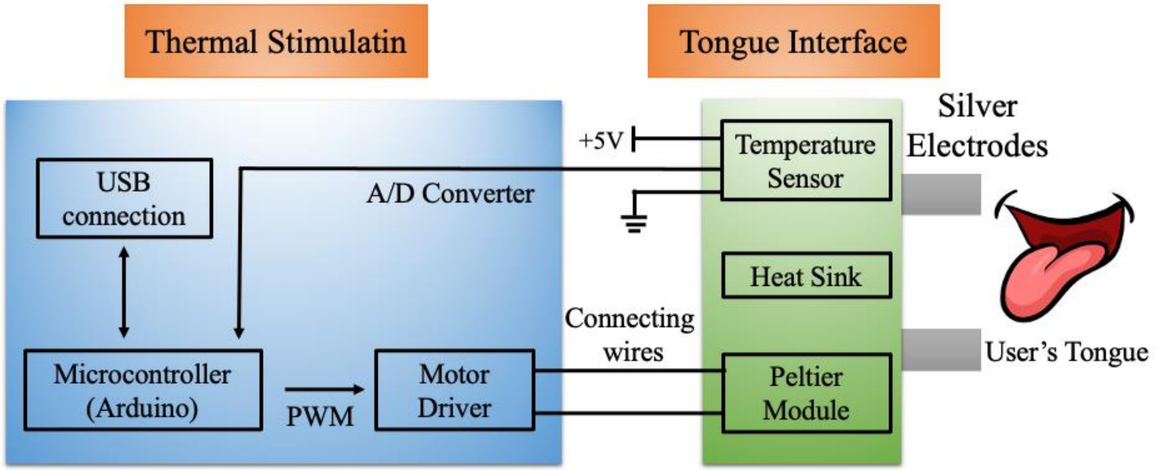

Figure S2. The thermal stimulation subsystem includes a motor driver to drive the current in two paths. The motor driver heat/ cold the Peltier module by varying the current path.

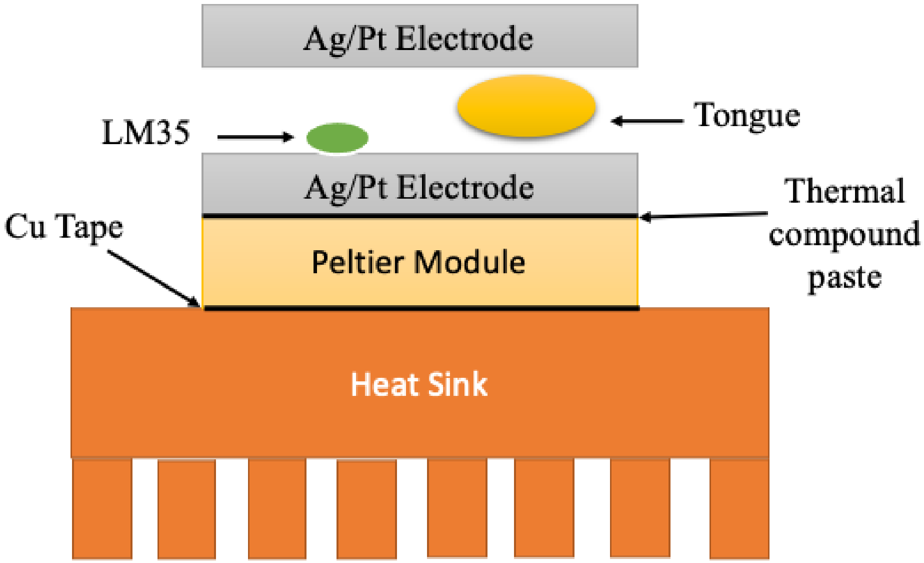

Figure S3. The tongue interface includes two silver electrodes with platinum plating attached to the Peltier module and LM35 temperature sensor. In contrast, the Peltier module is connected to a more oversized heatsink to cool it down.

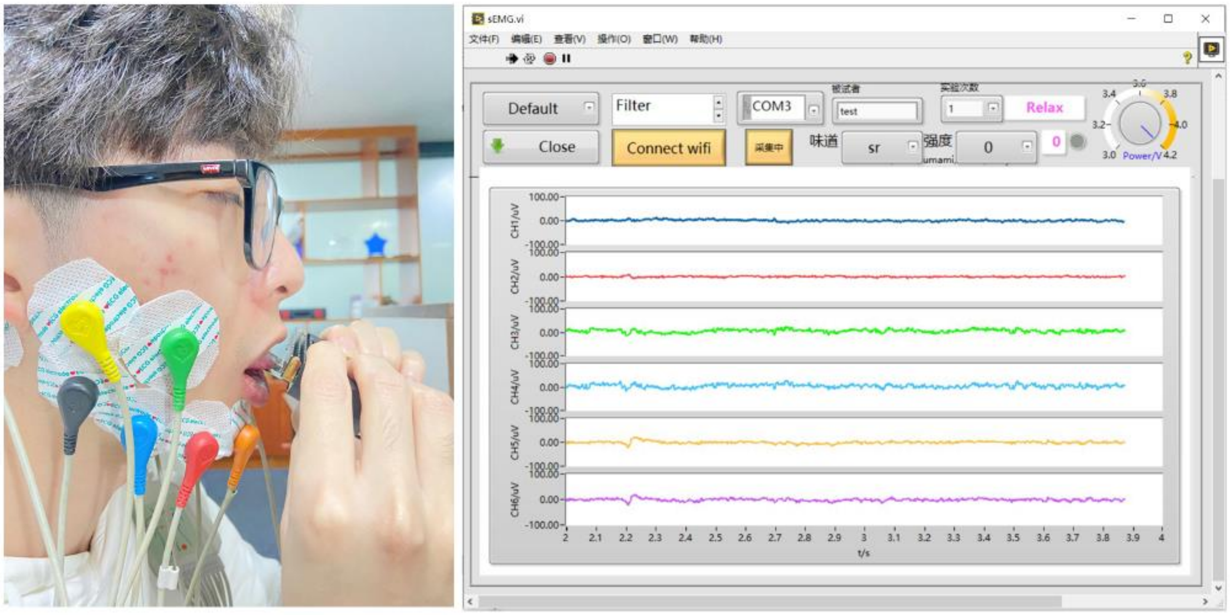

(a) (b)

Figure S4. Data acquisition diagram (a) Silver electrodes of sEMG are placed on facial muscles while silver electrodes of E-Taste are placed on the tip of the tongue (b) sEMG acquisition signals shown in LabVIEW human-computer interface.

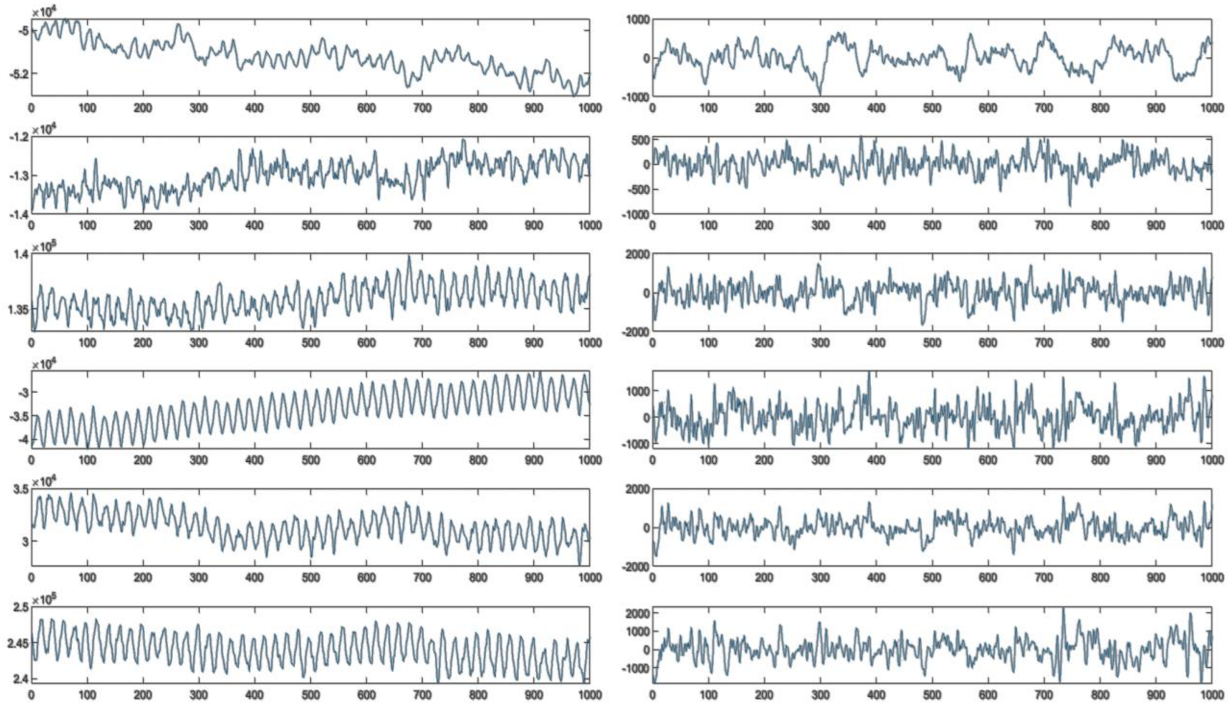

Figure S5. Schematic diagram of standard signals (within the range of -2000 μV to 2000 μV) after preprocessing.

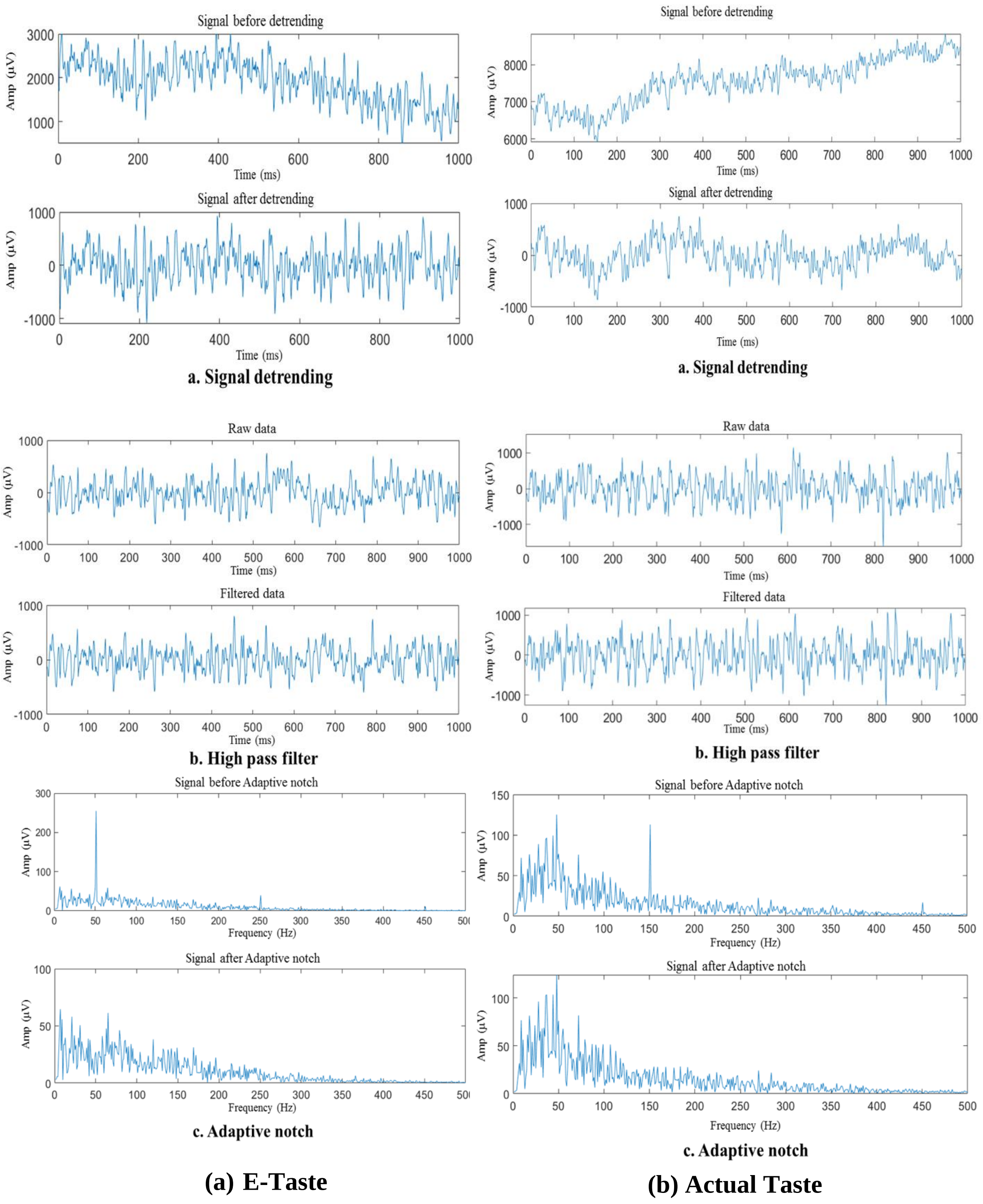

Figure S6. Preprocessing illustration of **(a)** E-Taste states and **(b)** Actual taste states.
